# Supplementary figures and images for: Early identification of high-risk individuals for mortality after lung transplantation: A retrospective cohort study with topological feature engineering
Source: PLOS Digit Health. 2026 May 5;5(5):e0001050. doi: 10.1371/journal.pdig.0001050 (PMC13143088; doi:10.1371/journal.pdig.0001050)

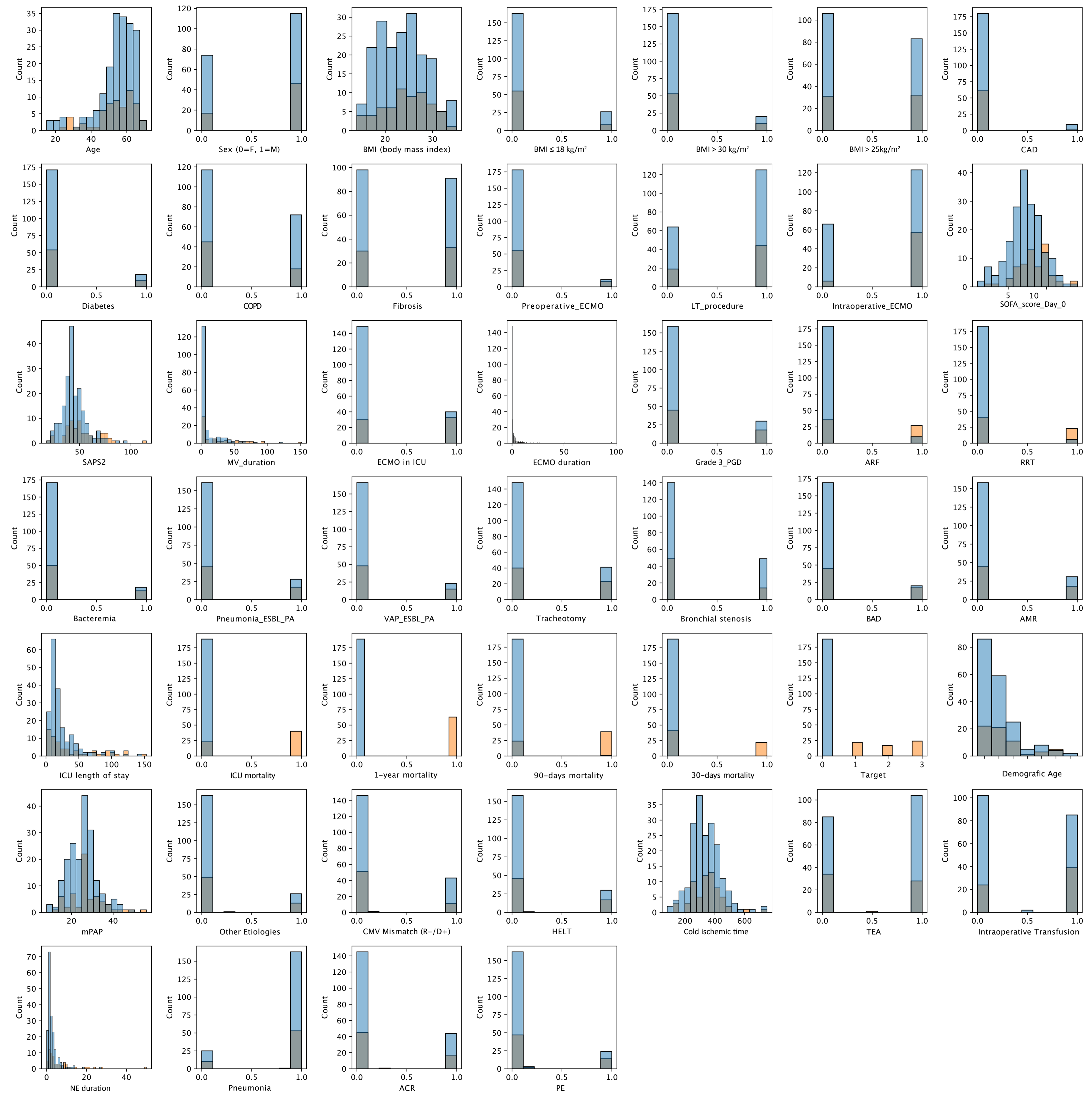

Supplement: S1 Fig — Histogram distributions of cohort variables are presented in each panel, illustrating the statistical analysis of patients categorized according to binary risk mortality. (PDF) [file pdig.0001050.s001.pdf]

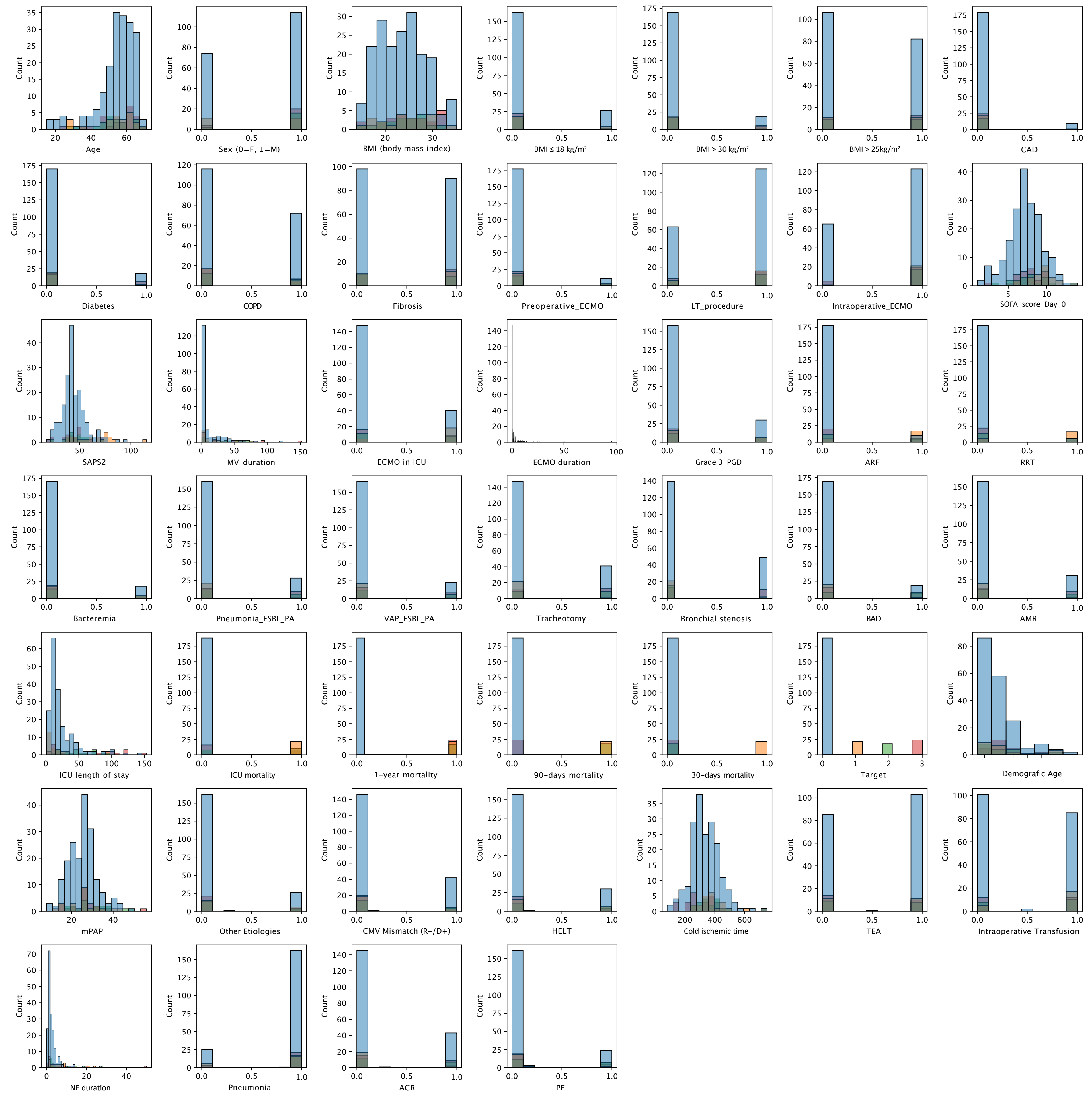

Supplement: S2 Fig — Histogram distributions of different variables are displayed in each panel, elucidating the statistical analysis of patients categorized based on “target scores” risk mortality. (PDF) [file pdig.0001050.s002.pdf]

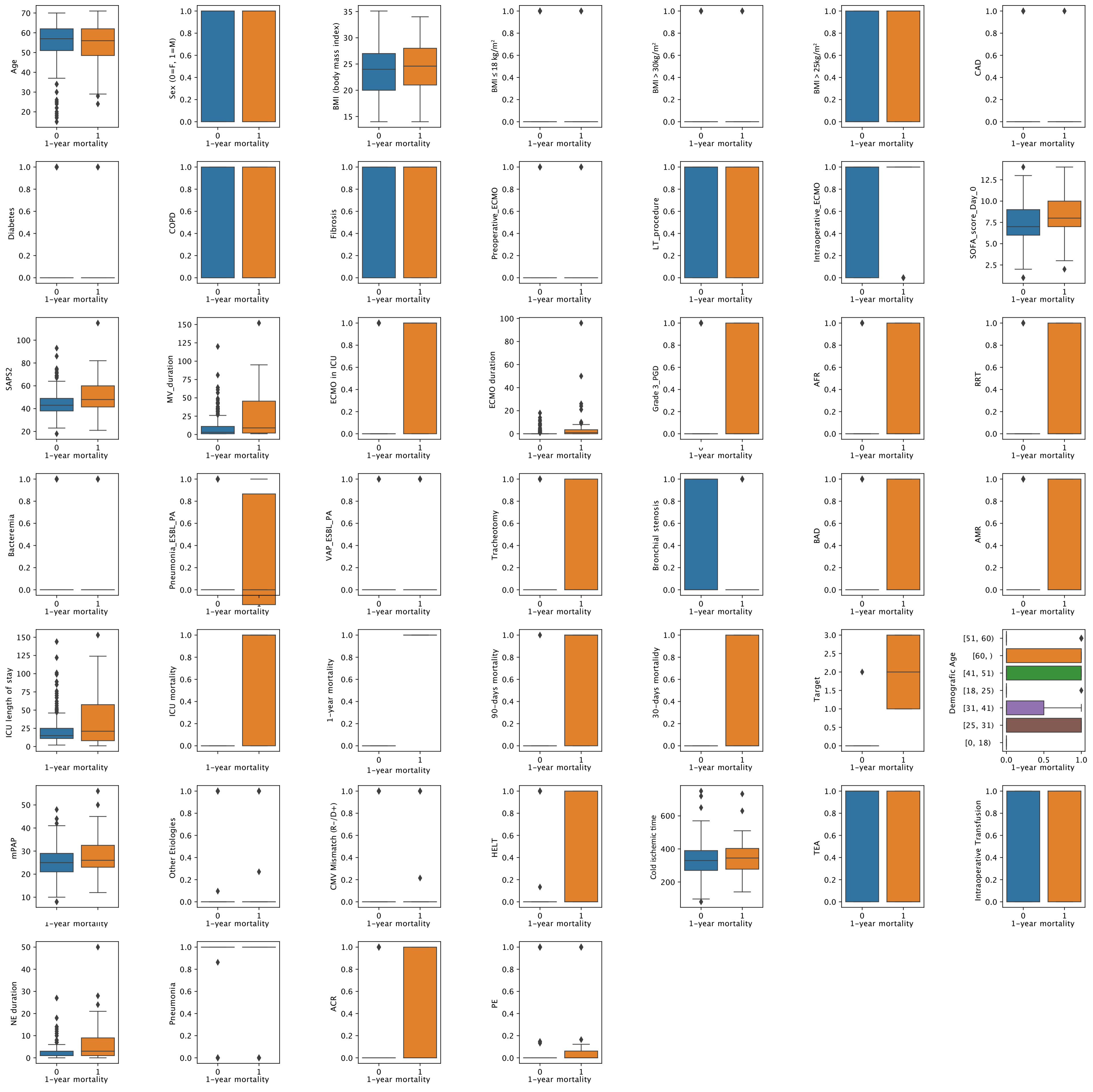

Supplement: S3 Fig — Boxplot distributions of respective variables are exhibited in each panel, providing insights into the statistical analysis of patients categorized by binary risk mortality. (PDF) [file pdig.0001050.s003.pdf]

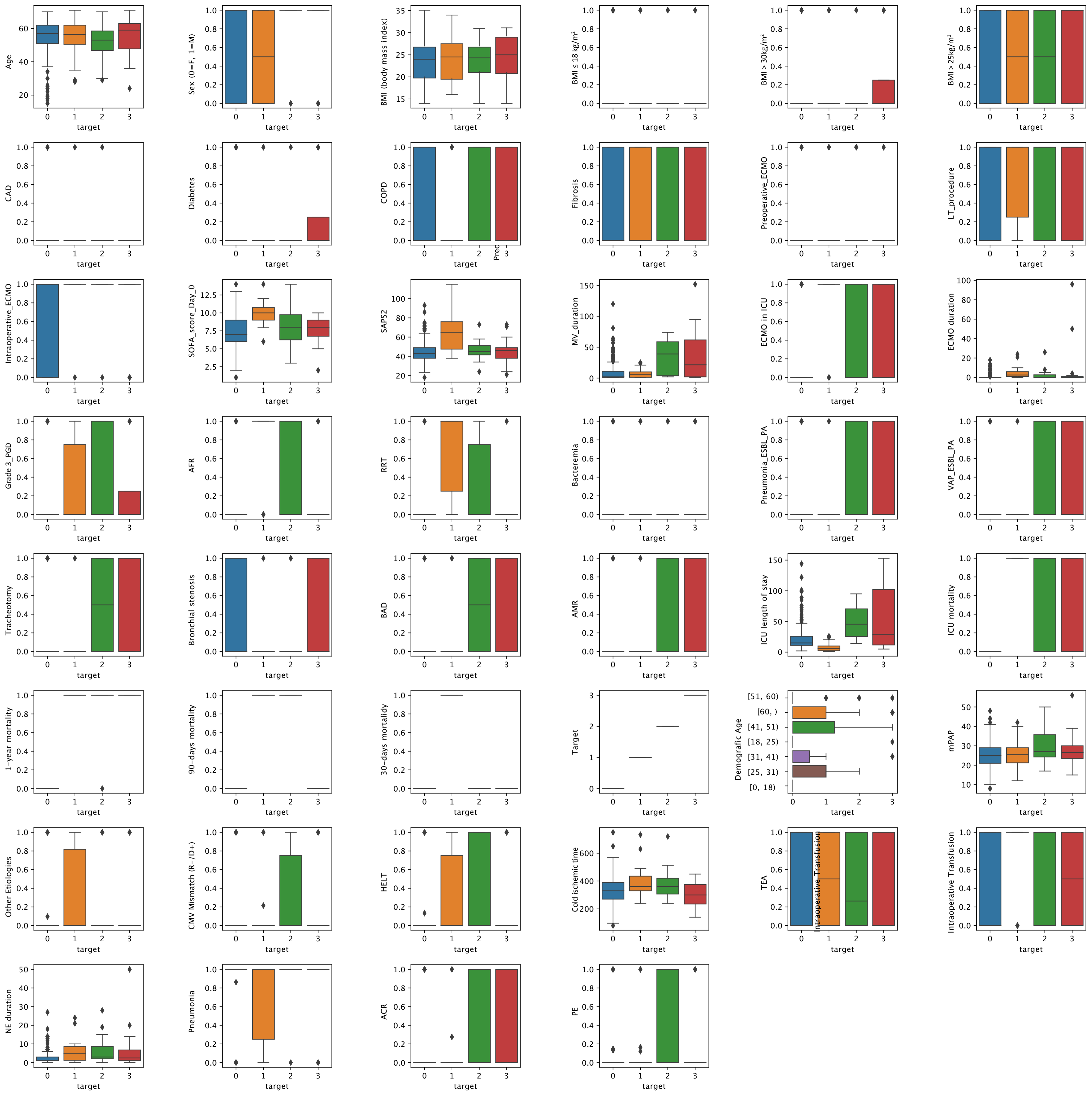

Supplement: S4 Fig — Boxplot distributions of corresponding variables are showcased in each panel, shedding light on the statistical analysis of patients categorized by “target scores” risk mortality. (PDF) [file pdig.0001050.s004.pdf]

Calibration Curve  
ECE = 0.080, Brier = 0.219

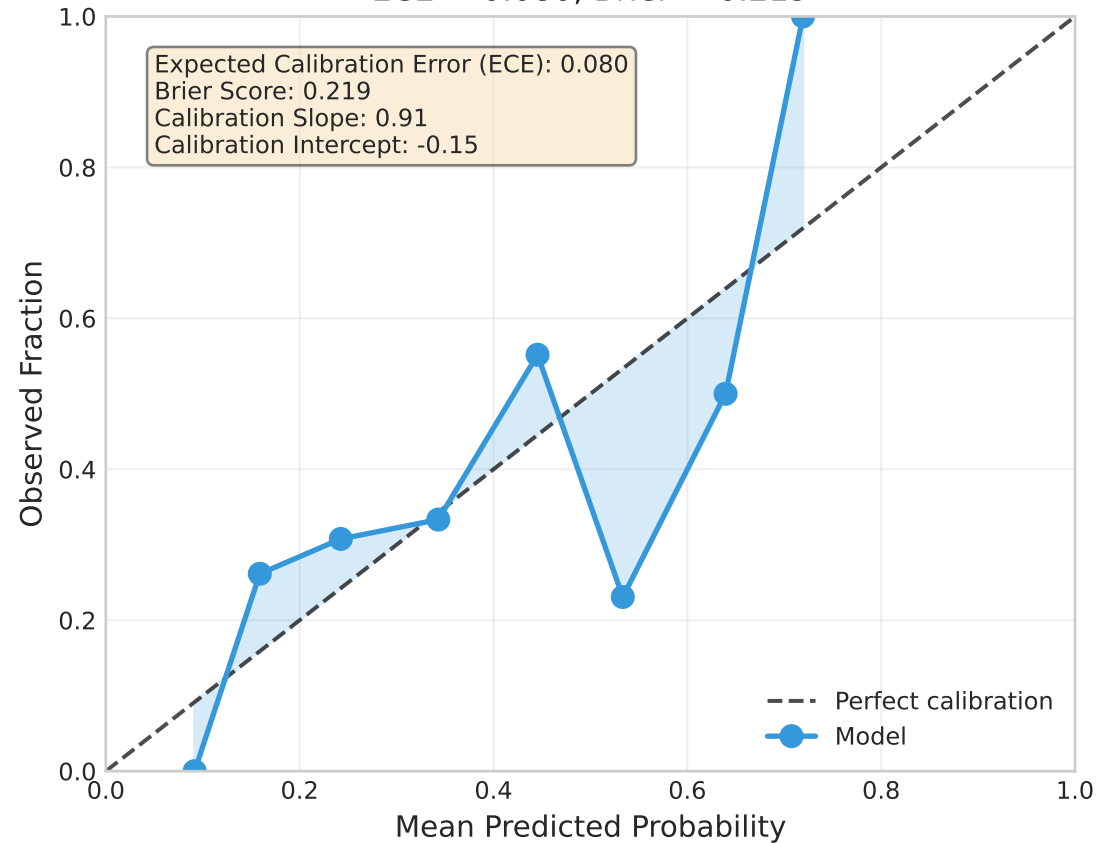

Reliability Diagram with Prediction Distribution

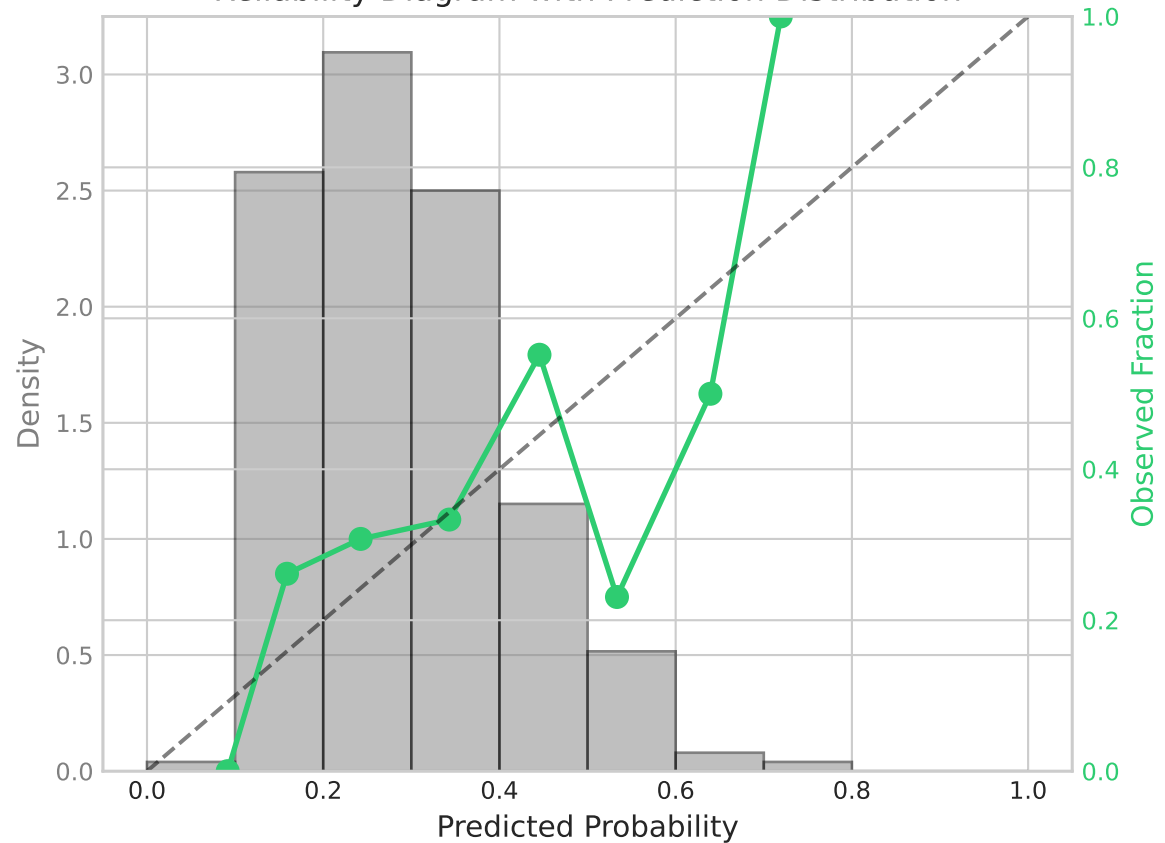

Supplement: S5 Fig — Left panel: Calibration curve showing predicted versus observed probabilities (ECE = 0.08, Brier score = 0.12). Right panel: The reliability diagram with prediction distribution (right panel) displays the histogram of predicted probabilities (gray bars) with the observed fraction of events (green line) across ten equally spaced bins. (PDF) [file pdig.0001050.s005.pdf]

# Decision Curve Analysis

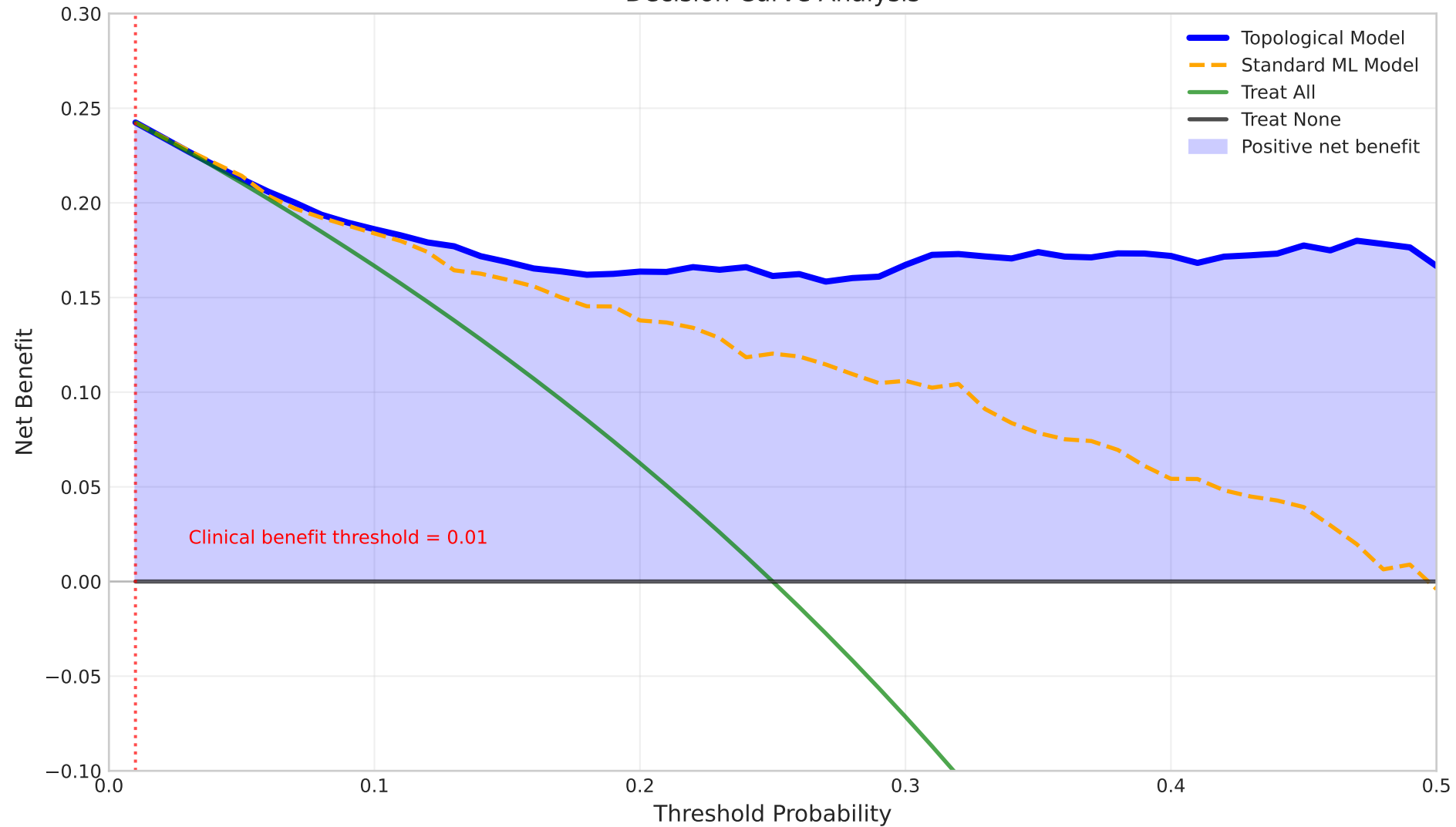

Supplement: S6 Fig — In panel a, we started with inpatient data and visualized the data records for each co-accessible peak per lung recipient over time. We then used a non-linear manifold to represent this data in a way that is accessible to medical users. Next, in computation step 1, we identified important clinical latent variables and encoded them using a single vector of topological extractors that maintain the structure of the data. We used this topological vector as an estimator in a machine learning predictor to predict mortality risk in computation step 2. Additionally, we were able to use the topological extractors to track cohort risk factor trajectories dynamically. Finally, we predicted the impact of each variable and quantified their interaction effects on the risk model of Y1 mortality. Based on the interpretability of the obtained learning model, we assigned a risk score to each patient, providing a valuable tool for clinicians in managing the care of lung transplant recipients. b, shows the construction of a low-dimensional embedding of data patient while clusters samples by a diffusion process that fits well the spotted branching trajectories of our data. We transform clinical variables into diagrams of persistence visualized by densities and localize the most suitable candidates to be homology generators of dimension 0, 1, and 2 in data patient. In panel c, the homology generators are used as tiled regions where to extract important clinical latent variables. We show a frequent situation of image persistence extractor at different pixel resolutions for survivor and non-survivor patients on the left and right, respectively. d, shows the trajectory inference between survivor and non-survivor clusters. e, visualizes transformed data patients by the picked topological transformer using the matrix of affinity calculated after applying the non-linear learning manifold technique. A smooth version of the data can be retrieved after affinity application, as shown in panel f, w [file pdig.0001050.s006.pdf]

a

Input data

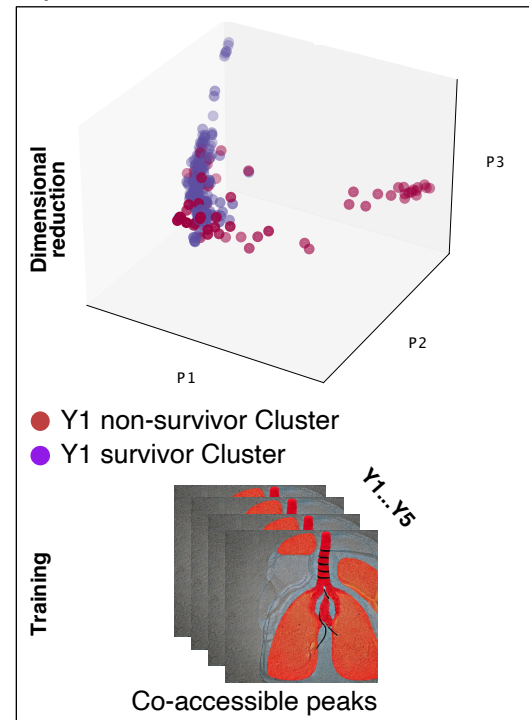

Computation step 1

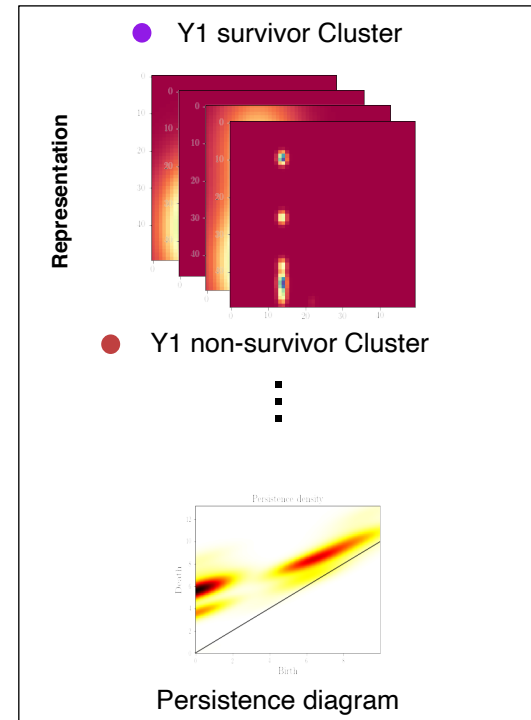

Computation step 2

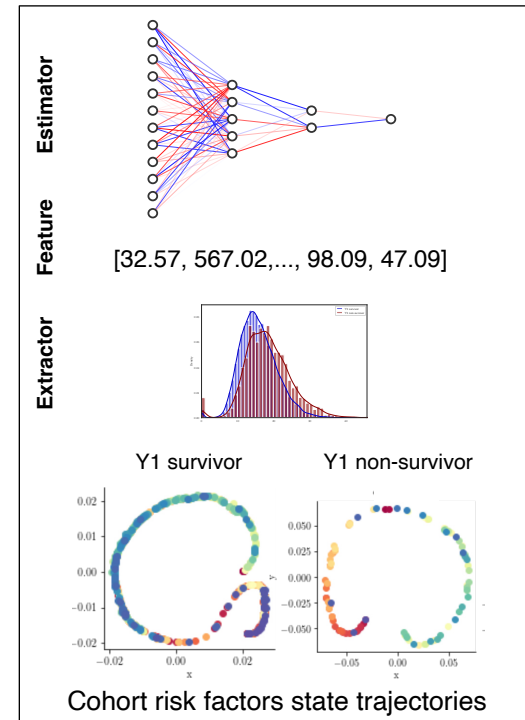

Output

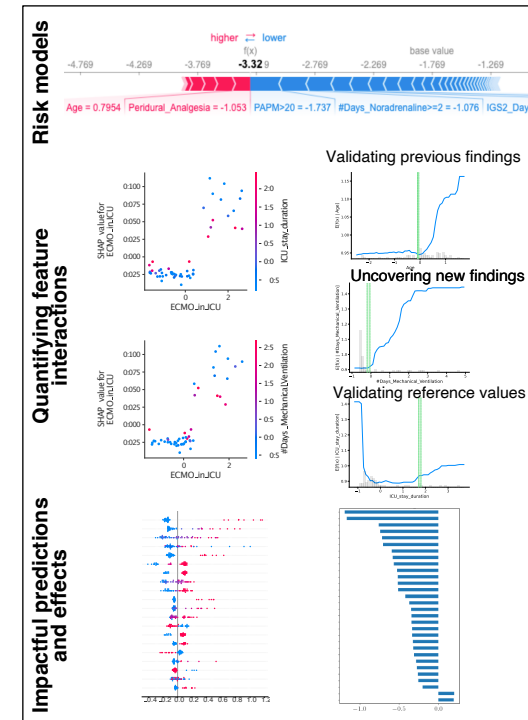

b

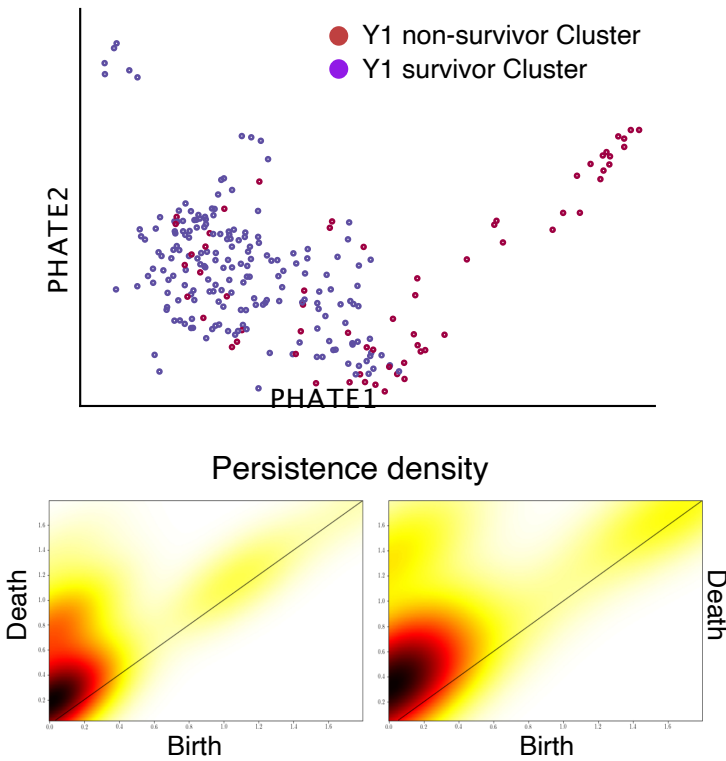

c

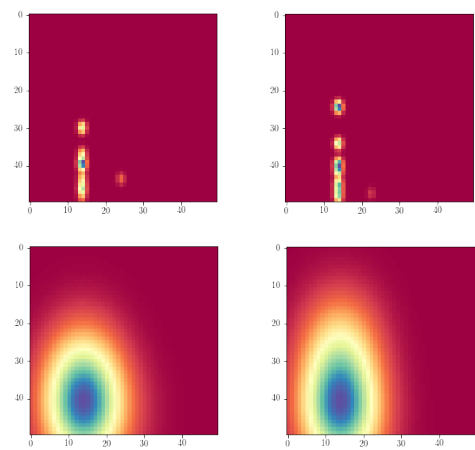

e

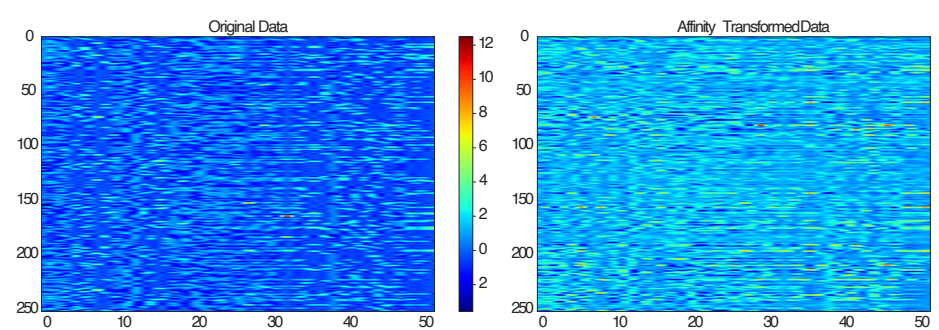

g

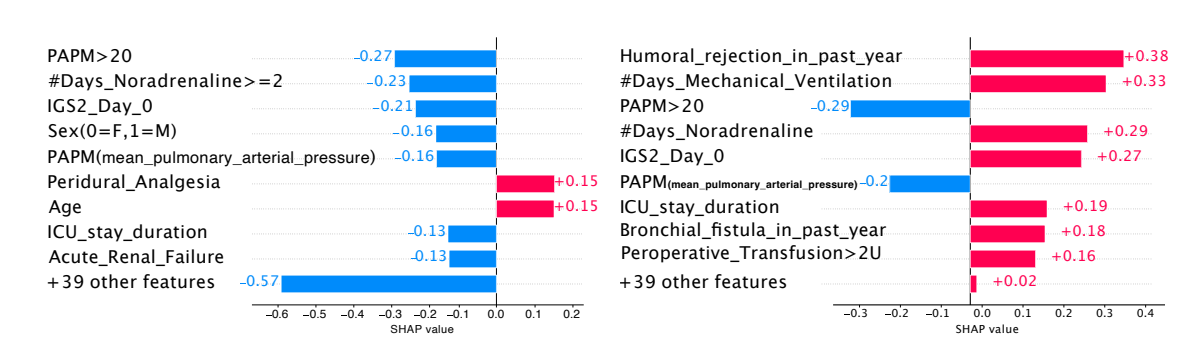

f

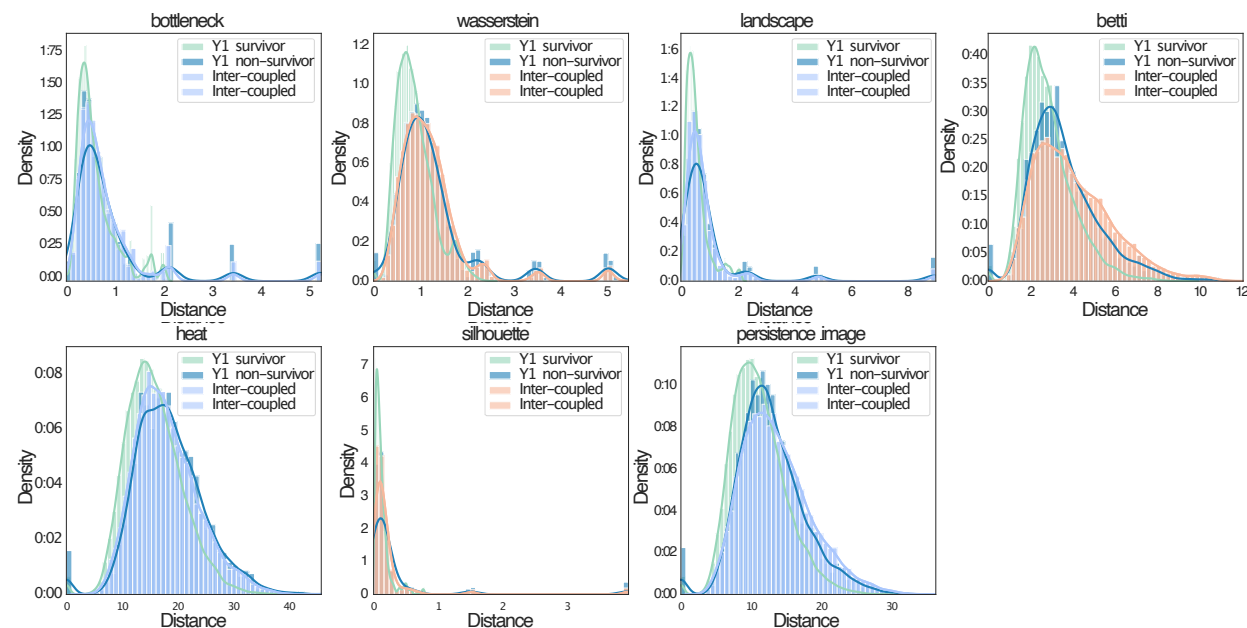

h

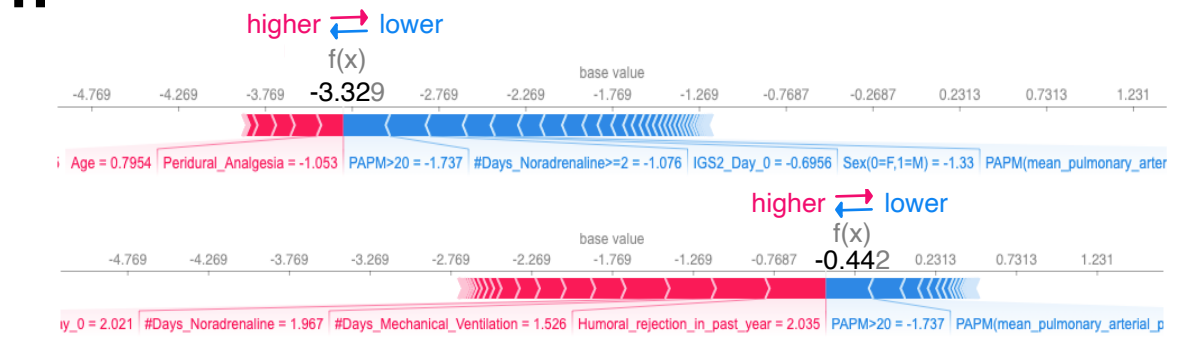

i

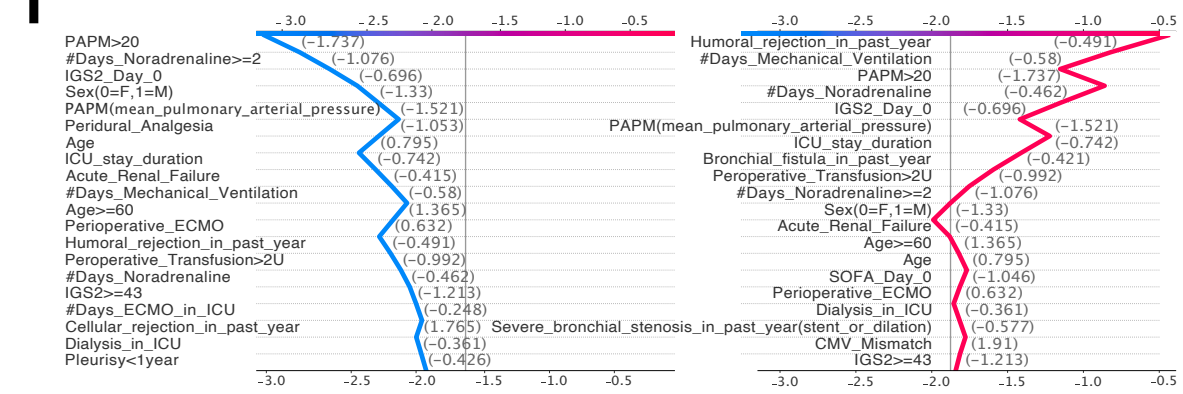

d

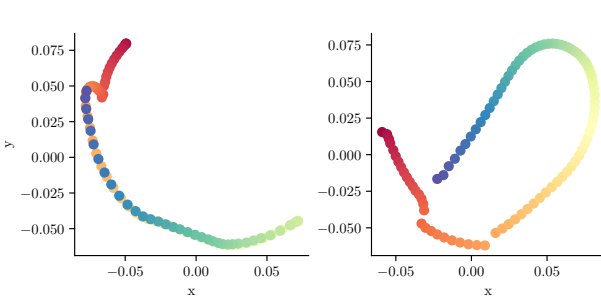

Supplement: S7 Fig — Heatmap of the top 10 features individually, while grouping the remaining 39. Positive values are typically shown in shades of red, while negative values are represented in shades of blue. (PDF) [file pdig.0001050.s007.pdf]

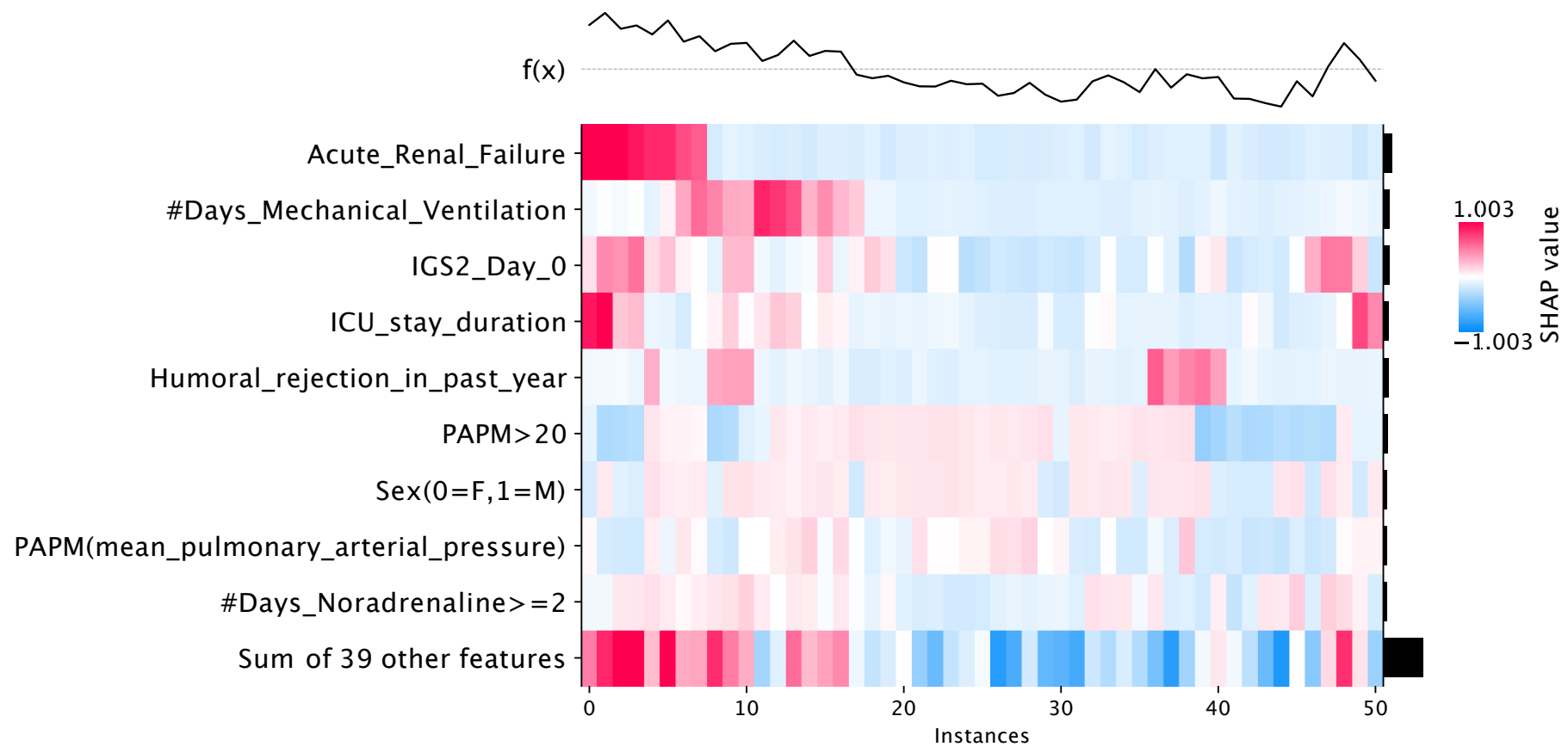

Supplement: S8 Fig — SHAP force plot that describes the feature contributions to the model prediction for each individual instance in the cohort. (PDF) [file pdig.0001050.s008.pdf]

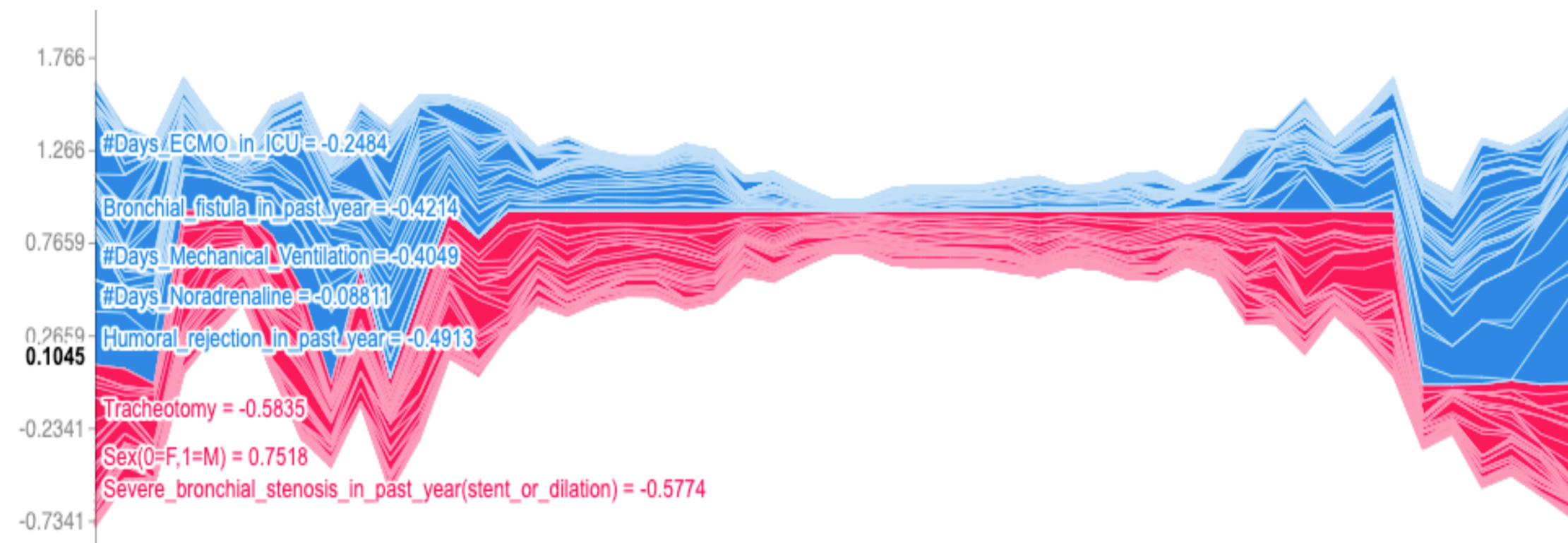

Supplement: S9 Fig — a) SHAP dependence plot illustrating the local feature interactions for the top 3 most impactful variables at only one different interacting feature value each time. b) SHAP dependence plot for ICU stay duration, showing a complex relationship with interacting features. (PDF) [file pdig.0001050.s009.pdf]

**b**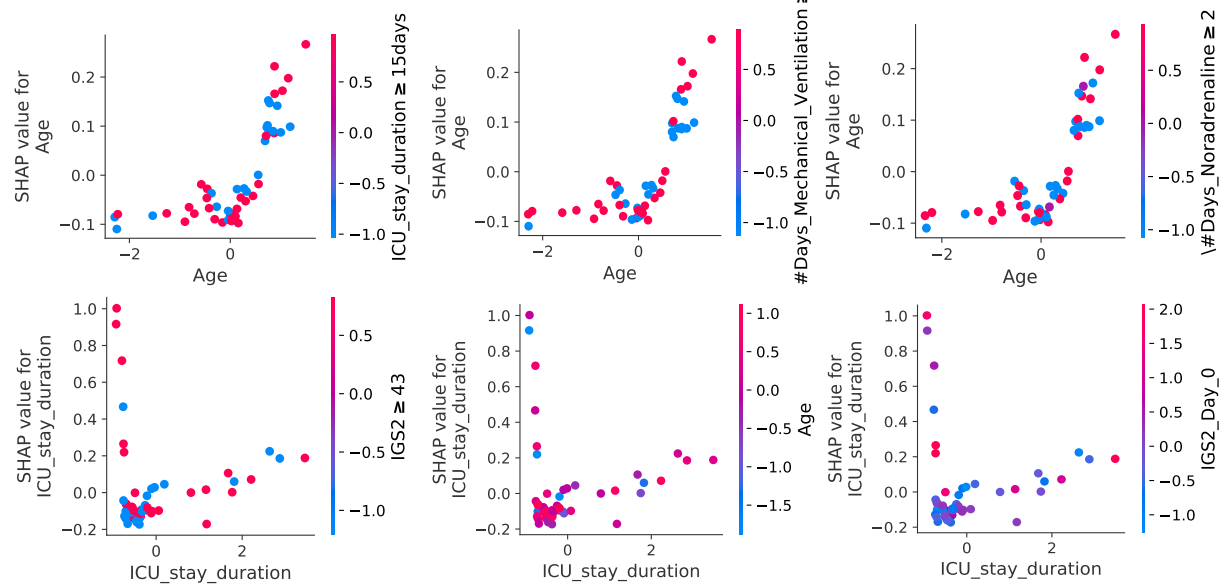

Supplement: S10 Fig — Net benefit of the topological model (blue line) compared to treat-all (green), treat-none (black), and standard ML (orange) strategies. The model provides clinical benefit for threshold probabilities >0.15, with positive net benefit indicated by the blue shaded region. (PDF) [file pdig.0001050.s010.pdf]
