# Supplementary material for: Early identification of high-risk individuals for mortality after lung transplantation: A retrospective cohort study with topological feature engineering
Source: PLOS Digit Health. 2026 May 5;5(5):e0001050. doi: 10.1371/journal.pdig.0001050 (PMC13143088; doi:10.1371/journal.pdig.0001050)
Supplement: S1 Text — Detailed description of the hyperparameters and software settings used for topological feature extraction and dimensionality reduction. (PDF) [file pdig.0001050.s015.pdf]

Supplementary Methods S1: Topological Data Analysis and PHATE Parameters

1. PHATE (Potential of Heat-diffusion for Affinity-based Transition Embedding)

Implementation Details
- **Software**: `phate` Python package v1.0.11
- **Reference**: Moon et al. (2019) Nature Biotechnology

Parameter Selection

Parameter	Value	Selection Method	Description
`k-neighbors`	5	Grid search {3,5,7,10} based on embedding quality	Number of nearest neighbors for graph construction
`decay`	15	Default	Decay rate for adaptive anisotropic kernel
`t`	'auto'	Knee point analysis of diffusion operator	Diffusion time scale
`n_landmark`	2000	Based on dataset size (252 patients)	Number of landmark points for approximation
`gamma`	1	Default	Decay constant for Gaussian kernel
`knn_dist`	'euclidean'	After standardization	Distance metric for kNN graph
`mds_dist`	'euclidean'	Default	Distance metric for MDS initialization
`mds`	'metric'	Default	Type of MDS (metric vs non-metric)
`random_state`	42	Fixed for reproducibility	Random seed
`n_jobs`	-1	Default	Number of parallel jobs

Preprocessing Steps
1. Standardize all continuous variables to mean=0, std=1
2. Handle missing values via MICE imputation before PHATE
3. Fit PHATE on training data only, transform validation/test sets

Quality Metrics
- **Stress**: 0.12 (final embedding)
- **Trustworthiness**: 0.89 (10 neighbors)
- **Continuity**: 0.91 (10 neighbors)

---

2. Persistent Homology

Implementation Details
- **Software**: GUDHI v3.7.0, giotto-tda v0.5.1
- **References**: Carrière et al. (2020), GUDHI Project

Filtration Parameters

Parameter	Value	Selection Method	Description
**Filtration type**	Vietoris-Rips	Standard for point cloud

data | Constructs simplices based on pairwise distances |
**Max edge length**	2.0	95th percentile of pairwise distances	Maximum filtration value
**Homology dimensions**	0, 1, 2	Based on data complexity	H0: components, H1: loops, H2: voids
**Coefficient field**	Z/2Z	Default	Field for homology computation
**Min persistence**	0.01	Default	Minimum persistence to consider

Persistence Diagram Parameters

Parameter	Value	Description
**Diagram resolution**	100×100 pixels	Grid size for persistence images
**Spread**	1.0	Standard deviation for Gaussian weighting
**Weight function**	Gaussian (σ=0.5)	Weight = persistence × Gaussian(birth)
**Weight range**	[0, 1]	Normalized weight values

Persistence Image Generation

For each persistence diagram, we generate images as:

```python
def persistence_image(diagram, resolution=100, spread=1.0, sigma=0.5):
    '''Convert persistence diagram to image'''
    image = np.zeros((resolution, resolution))

    for birth, death in diagram:
        persistence = death - birth
        if persistence > 0:
            # Gaussian weight centered at birth
            x = np.linspace(0, 1, resolution)
            y = np.linspace(0, 1, resolution)
            X, Y = np.meshgrid(x, y)

            weight = persistence * np.exp(-((X - birth) ** 2) / (2 * sigma ** 2))
            image += weight

    return image / np.max(image)  # Normalize
```
