# Supplementary material for: Early identification of high-risk individuals for mortality after lung transplantation: A retrospective cohort study with topological feature engineering
Source: PLOS Digit Health. 2026 May 5;5(5):e0001050. doi: 10.1371/journal.pdig.0001050 (PMC13143088; doi:10.1371/journal.pdig.0001050)
Supplement: S2 Text — Step-by-step description of the cross-validation strategy, test set splitting, baseline model definitions, and statistical testing procedures. (PDF) [file pdig.0001050.s016.pdf]

Supplementary Methods S2: Complete Evaluation Protocol

1. Nested Cross-Validation Strategy

Outer Loop (5-fold)

Purpose: Unbiased performance estimation

Split: Stratified by outcome (maintains 75%/25% distribution)

Process:

Each fold: 80% training+validation, 20% test

No test data used in any training decisions

Performance reported as mean ± SD across 5 test folds

Inner Loop (3-fold)

Purpose: Hyperparameter tuning

Split: Further split of training data (80% train, 20% validation)

Process:

Grid search over hyperparameter space

Best parameters selected based on mean validation AUC

Retrained on full training set for outer fold evaluation

Leakage Prevention

All preprocessing (scaling, imputation) fitted only on training folds

Transform parameters saved and applied to validation/test folds

Feature selection performed within inner loop only

No information from test sets used at any stage

2. Hyperparameter Tuning Grid

MLP Architecture

Parameter	Values Tested	Selected
Hidden layer sizes	(64,32), (128,64), (32,16)	(64,32)
Activation	['relu', 'tanh']	'relu'
Dropout rate	[0.1, 0.2, 0.3]	0.2
L2 regularization (α)	[0, 0.001, 0.01, 0.1]	0.01
Learning rate	[0.001, 0.01, 0.1]	0.001
Batch size	[16, 32, 64]	32
Max iterations	[100, 200, 500]	200
Early stopping patience	[5, 10, 15]	10

Training Parameters

Parameter	Value
Optimizer	Adam (β1=0.9, β2=0.999, ε=1e-8)
Learning rate schedule	Constant
Validation fraction	0.2 (of training data)

Class weights	{0: 1.0, 1: 3.0} (inverse frequency)
Random seed	42 (fixed for reproducibility)

3. Performance Metrics

Primary Metrics (with 95% CI from 2000 bootstrap samples)

Metric	Formula	Threshold
Accuracy	(TP + TN) / N	Youden's index
Sensitivity (Recall)	TP / (TP + FN)	Youden's index
Specificity	TN / (TN + FP)	Youden's index
PPV (Precision)	TP / (TP + FP)	Youden's index
NPV	TN / (TN + FN)	Youden's index
F1-score	2 × (Precision × Recall) / (Precision + Recall)	Youden's index
ROC-AUC	Area under ROC curve	Threshold-independent
PR-AUC	Area under precision-recall curve	Threshold-independent

Calibration Metrics

Metric	Formula	Value
Brier score	$(1/N) \Sigma (y\_pred - y\_true)^2$	0.12
ECE	Σ P(k) - O(k) × (n_k / N)	0.08
Calibration slope	β from logit(y_true) ~ β × logit(y_pred)	0.91
Calibration intercept	α from logit calibration	-0.15

Decision Curve Analysis

Net benefit at threshold t:

Net Benefit = (TP/N) - (FP/N) × (t/(1-t))

4. Bootstrap Confidence Intervals

Procedure for each metric:

For each outer fold test set (n ≈ 50):

Resample with replacement 2000 times

Maintain stratification by outcome

Calculate metric on each bootstrap sample

Calculate 2.5th and 97.5th percentiles for 95% CI

Report mean across folds ± bootstrap SE

5. Statistical Tests

Comparison	Test	Adjustment
Model vs baseline	DeLong test for AUC	-
Subgroup heterogeneity	Cochran's Q test	-
Feature importance	Cohen's d with 95% CI	BH-FDR q<0.05
Paired comparisons	Paired t-test (cross-validation folds)	-

6. Software and Packages

Package	Version	Purpose
Python	3.9.0	Core language
numpy	1.21.0	Numerical computing
pandas	1.3.0	Data manipulation
scikit-learn	1.0.0	Machine learning
phate	1.0.11	Manifold learning
giotto-tda	0.5.1	Topological analysis

GUDHI	3.7.0	Persistent homology
shap	0.40.0	Model interpretability
matplotlib	3.4.0	Visualization
seaborn	0.11.0	Statistical visualization
scipy	1.7.0	Statistical tests

7. Reproducibility Checklist

Random seeds fixed (42)

Nested cross-validation implemented

Preprocessing fitted on training only

Hyperparameters selected via inner CV

Bootstrap CIs for all metrics

Calibration assessment

Decision curve analysis

Subgroup analyses

Effect sizes with confidence intervals

Multiple comparison correction (BH-FDR)

Code publicly available

Synthetic data for validation

8. Code Availability

```
git clone https://github.com/MorillaLab/TopoTransformers
cd TopoTransformers
conda env create -f environment.yml
conda activate topo_lung
python run_analysis.py --seed 42 --cv 5 --bootstrap 2000
```
