# Supplementary material for: Early identification of high-risk individuals for mortality after lung transplantation: A retrospective cohort study with topological feature engineering
Source: PLOS Digit Health. 2026 May 5;5(5):e0001050. doi: 10.1371/journal.pdig.0001050 (PMC13143088; doi:10.1371/journal.pdig.0001050)
Supplement: S3 Text — Summary of model architecture, intended use cases, input features, training data characteristics, performance benchmarks, and known limitations. (PDF) [file pdig.0001050.s017.pdf]

MODEL CARD: Topological Feature Model for Lung Transplantation Mortality Prediction

Model Details
- **Model Name**: TopoTransplant-v1
- **Version**: 1.0.0
- **Date**: March 2025
- **Model Type**: Multi-Layer Perceptron with topological feature extraction
- **Developed by**: Tran-Dinh, Morilla et al., Bichat Hospital / Université Paris Cité
- **License**: Academic use only (contact authors for commercial use)

Architecture
- **Input features**: 76 (49 clinical + 27 topological features)
- **Hidden layers**: 2 layers (64, 32 neurons)
- **Activation functions**: ReLU (hidden), Sigmoid (output)
- **Dropout**: 0.2 after each hidden layer
- **Regularization**: L2 ($\lambda=0.01$)
- **Optimizer**: Adam (learning rate = 0.001)
- **Loss function**: Binary cross-entropy with class weights

Topological Feature Extraction
- **Method**: Persistent homology with Vietoris-Rips filtration
- **Homology dimensions**: 0, 1, 2
- **Feature vectorization**: Persistence images (100×100 pixels)
- **Features per dimension**: 9 (3 extractors × 3 metrics)
- **Total topological features**: 27

Intended Use
- **Primary use**: Risk stratification for one-year mortality after lung transplantation
- **Clinical setting**: Post-operative ICU and transplant follow-up
- **Target population**: Adult lung transplant recipients
- **Not intended for**:
  - Multi-organ transplant recipients
  - Retransplantation cases
  - Standalone clinical decision-making without physician oversight
  - Prediction beyond 1-year post-transplant

Factors
Demographic Factors Considered
- Age (continuous)
- Sex (binary)
- Body mass index (continuous)

Clinical Factors Considered
- Pre-operative: Disease etiology, comorbidities, pulmonary pressure, ECMO
- Intra-operative: Transplant type, ischemia time, transfusion, ECMO
- Post-operative: ICU variables (SOFA, SAPS2), organ support (ECMO, MV, vasopressors), complications (PGD, ARF, rejection), infections

Evaluation Factors
- Model performance evaluated across:
  - Age groups (<50, ≥50 years)
  - Sex (male, female)
  - Disease etiology (COPD, ILD, CF, other)
  - Transplant type (single, double)

Metrics
Overall Performance (95% CI)
Metric	Value
Accuracy	0.874 (0.831-0.917)
Sensitivity	0.841 (0.762-0.920)
Specificity	0.896 (0.851-0.941)
PPV	0.817 (0.734-0.900)
NPV	0.912 (0.871-0.953)
F1-score	0.829 (0.758-0.900)
ROC-AUC	0.870 (0.810-0.930)
PR-AUC	0.710 (0.620-0.800)
Brier score	0.12
ECE	0.08

Subgroup Performance
Subgroup	n	AUC (95% CI)
Age <50	98	0.86 (0.78-0.94)
Age ≥50	154	0.88 (0.81-0.95)
Male	158	0.87 (0.80-0.94)
Female	94	0.86 (0.78-0.94)
COPD	89	0.89 (0.82-0.96)
ILD	78	0.86 (0.78-0.94)
CF	42	0.84 (0.74-0.94)
Single LT	67	0.84 (0.76-0.92)
Double LT	185	0.88 (0.81-0.95)

Training Data
- **Source**: Bichat Hospital, Paris (2015-2020)
- **Sample size**: 252 patients (189 survivors, 63 non-survivors)
- **Splits**: 5-fold nested cross-validation
- **Class imbalance handling**: Inverse frequency class weights (1:3 ratio)

Evaluation Data
- **Validation strategy**: Nested cross-validation (outer 5-fold, inner 3-fold)
- **Test sets**: Held-out folds (20% per fold)
- **Total test samples**: 252 (across all folds)

Ethical Considerations
- **Privacy**: All patient data anonymized; synthetic data provided for code validation
- **Bias**: Subgroup analysis showed no significant performance disparities, though power limited for small subgroups

- **Fairness**: Model should not be used for resource allocation decisions without further validation
- **Transparency**: SHAP analysis provided for interpretability; model not a black box
- **Clinical integration**: Intended as decision support, not autonomous decision-making

Deployment Metrics

Metric	Value
Parameters	15,842
FLOPs per inference	3.2M
Inference latency (CPU)	12 ms
Inference latency (GPU)	<1 ms
Memory footprint	64 MB
Model size (serialized)	632 KB

Limitations
- Single-center retrospective design
- Modest sample size (63 events)
- Requires external validation
- Postoperative variables not available at transplantation (requires sequential updating)
- Potential for overfitting despite nested CV
- Not validated for retransplantation or multi-organ transplant

Maintenance
- **Version control**: GitHub repository with version tags
- **Updates**: Model will be updated with new data; versioning maintained
- **Contact**: ian.morilla@uma.es for questions

References
- Mitchell et al. (2019) "Model Cards for Model Reporting"
- Tran-Dinh, Morilla et al. (2025) PLOS Digital Health (in review)

This model card was generated on March 1, 2025
