# Supplementary material for: Early identification of high-risk individuals for mortality after lung transplantation: A retrospective cohort study with topological feature engineering
Source: PLOS Digit Health. 2026 May 5;5(5):e0001050. doi: 10.1371/journal.pdig.0001050 (PMC13143088; doi:10.1371/journal.pdig.0001050)
Supplement: Attachment — Submitted filename: Response_to_Reviewers_auresp_2.pdf [file pdig.0001050.s020.pdf]

Additional Editor Comments (if provided):

Thanks for the response and the revision. Please address the remaining reviewer concerns. Moreover, I did not find any response to my comments -- please address all of them too. Furthermore, prepare the response cleanly by colour coding the comments and responses so that it is easy to read.

>> **Reply:** We acknowledge this serious concern (release of patient information on the authors' GitHub repository) and have taken immediate and comprehensive action as described in the response to reviewers throughout R1. We have also expanded the ethics statement to include complete approval details, reference number, and legal basis. We have reviewed the TRIPOD-AI checklist and ensured that our manuscript now includes all recommended elements, with particular emphasis on calibration reporting. We have added:

1. Explicit calibration metrics in Results (ECE, Brier score, calibration slope and intercept)
2. Calibration curves (Figure S5) with detailed description
3. TRIPOD-AI checklist as supplementary material (Table S5)

"Finally, the conclusion states that the proposed model could be used for precision medicine and clinical decision support. While such a prognostic model may indeed assist with administrative planning and resource allocation, it cannot be used to guide treatment or intervention decisions aimed at reducing mortality risk. This limitation is well established in the causal inference literature. The authors are therefore advised to revise the wording accordingly."

>> **Reply:** We thank the Editor for this important distinction. We have carefully revised all statements throughout the manuscript to clarify that the model provides prognostic information for risk stratification and resource allocation, not causal guidance for treatment decisions. Key changes included throughout the R1 and R2.

Reviewers' Comments:

Reviewer's Responses to Questions
Comments to the Author

1. If the authors have adequately addressed your comments raised in a previous round of review and you feel that this manuscript is now acceptable for publication, you may indicate that here to bypass the "Comments to the Author" section, enter your conflict of interest statement in the "Confidential to Editor" section, and submit your "Accept" recommendation.

Reviewer #1: All comments have been addressed

Reviewer #3: All comments have been addressed

---

2. Does this manuscript meet PLOS Digital Health's publication criteria? Is the manuscript technically sound, and do the data support the conclusions? The manuscript must describe methodologically and ethically rigorous research with conclusions that are appropriately drawn based on the data presented.

Reviewer #1: Yes
Reviewer #3: Yes

---

3. Has the statistical analysis been performed appropriately and rigorously?

Reviewer #1: Yes
Reviewer #3: Yes

---

4. Have the authors made all data underlying the findings in their manuscript fully available (please refer to the Data Availability Statement at the start of the manuscript PDF file)?

The PLOS Data policy requires authors to make all data underlying the findings described in their manuscript fully available without restriction, with rare exception. The data should be provided as part of the manuscript or its supporting information, or deposited to a public repository. For example, in addition to summary statistics, the data points behind means, medians and variance measures should be available. If there are restrictions on publicly sharing data—e.g. participant privacy or use of data from a third party—those must be specified.
Reviewer #1: Yes
Reviewer #3: Yes

---

5. Is the manuscript presented in an intelligible fashion and written in standard English?

PLOS Digital Health does not copyedit accepted manuscripts, so the language in submitted articles must be clear, correct, and unambiguous. Any typographical or grammatical errors should be corrected at revision, so please note any specific errors here.

Reviewer #1: Yes
Reviewer #3: Yes

---

6. Review Comments to the Author

Please use the space provided to explain your answers to the questions above. You may

also include additional comments for the author, including concerns about dual publication, research ethics, or publication ethics. (Please upload your review as an attachment if it exceeds 20,000 characters)

Reviewer #1: good work

Reviewer #3: I appreciate the substantial effort the authors have invested in revising this manuscript. The paper is clearly improved relative to the prior version, and I want to acknowledge that many of the major methodological and presentation concerns have been addressed constructively. I also think the topic is clinically important, and the combination of topological feature engineering, dynamic postoperative data, and interpretable machine learning remains potentially valuable.

That said, a few issues still need attention before the manuscript is ready. Most of these are now in the category of clarification, calibration, and positioning rather than fundamental redesign, which is why I see this revision as close to acceptable with minor further revision. My comments below are aimed at helping the authors bring the paper fully to the level it should reach.

Comment 1: Page 1: The authors claim “This study introduces a novel predictive model based on topological transformers to assess mortality risk following lung transplantation.” I appreciate that the response letter states this terminology was corrected, but this phrasing still appears in the revised manuscript excerpt and should be removed fully and consistently. Since the implemented model is an MLP with topological feature engineering rather than a transformer architecture, I encourage the authors to ensure that the abstract, conclusion, keywords, and any residual figure labels use only accurate terminology throughout.

>> **Reply:** We thank the reviewer for catching this oversight. We have conducted a thorough review of the entire manuscript and ensured that all instances of "topological transformers" have been replaced with accurate terminology. The abstract, keywords, main text, figures, and figure legends now consistently use "topological feature engineering" or "topological feature-based prediction model."

Comment 2: Page 1: The authors claim “The proposed model demonstrated superior predictive performance compared to the Lung Transplant Risk Index and other benchmark models.” This statement is stronger than necessary in the abstract unless the fair baseline comparison is stated alongside it. Since the key methodological advance is not merely outperforming preoperative scores, but improving over standard ML models trained on the same postoperative feature set, I encourage the authors to mention that comparison explicitly in the abstract so the claim is both fairer and more informative.

>> **Reply:** We agree that the abstract should reflect the fair comparison context. We have revised the abstract to explicitly mention that our model outperforms both

traditional clinical scores and standard machine learning models trained on the same postoperative feature set.

Comment 3: Page 1–2: The authors claim “Shapley-based interpretability analysis revealed that dynamic variables such as early post-operative oxygenation trends, immunosuppressive load, and inflammatory markers were among the most critical contributors to mortality risk.” I think this is helpful, but the manuscript would still benefit from one short sentence clarifying whether these features are intended for sequential bedside updating rather than a one-time prediction model. The revised manuscript does address timing in the response letter, but that clinical use case should be visible more prominently in the main text because it resolves an important conceptual concern about “early identification.”

>> **Reply:** We have added explicit language in the abstract and introduction to clarify that the model supports sequential updating throughout the early postoperative course, enabling dynamic risk stratification rather than a single static prediction.

Comment 4: Page 3–4: The authors claim that current scores are limited because they are static and cannot capture evolving postoperative trajectories. I agree with this framing, and I think the revised introduction is stronger. However, I still encourage the authors to tighten the gap statement by making the novelty contrast more precise: the real contribution is not “machine learning after transplant” in general, but topological feature engineering applied to time-evolving post-transplant clinical trajectories, evaluated against fair non-topological baselines. That sharper wording would strengthen the manuscript’s positioning.

>> **Reply:** We have revised the introduction to sharpen the contrast and more precisely articulate the novel contribution.

Comment 5: Page 6–7: The authors claim they implemented a leakage-safe nested cross-validation strategy with preprocessing fitted only on training folds. This is an important and welcome improvement. I nevertheless encourage the authors to state directly in the main methods text, not only in the supplement or response letter, whether PHATE fitting and persistence image generation were recomputed within each outer training fold. This is implied, but it should be explicit because these steps are central to the novelty and to leakage prevention.

>> **Reply:** This is a constructive comment. We have added explicit language in the Methods section clarifying that all data transformations, including PHATE manifold learning and persistence image generation, were fitted exclusively on training folds and then applied to validation and test folds.

Comment 6: Page 7–9: The authors claim that 27 topological features were generated and then combined with 49 clinical variables. I appreciate that the revision now addresses overfitting more directly. However, because the event count remains modest, I encourage the authors to report in the main paper, not just in the response, the average number of features retained after RFECV across folds and whether the selected

topological features were stable across folds. That would make the feature-engineering story more convincing and reduce concern that performance depends on unstable selection.

>> **Reply:** Thank you for pointing this out! We have added this information to the Results section, including the average number of features retained and a measure of feature selection stability.

Comment 7: Page 10: The authors previously suggested that topological invariants help prevent overfitting. I appreciate that the response indicates this unsupported statement has been removed. I encourage the authors to make sure the revised manuscript is equally careful wherever topology is discussed, and to avoid language that implies topology itself replaces conventional regularization. The current paper is stronger when it presents topological features as informative representations rather than as a theoretical safeguard against overfitting.

>> **Reply:** We have reviewed all sections discussing topology and overfitting to ensure precise language. The text now clearly states that topological features serve as informative representations while standard regularization techniques (dropout, L2) are used for model training.

Comment 8: Page 14 and Results tables: The authors report strong overall performance. I think the revised inclusion of confidence intervals, PR-AUC, balanced metrics, and fair ML baselines materially improves the paper. One additional improvement would be to report the absolute gain over the best non-topological baseline directly in the main results narrative. At present, that important result is clearer in the response than in the manuscript. Since the topological contribution appears to be approximately a modest but real incremental AUC gain, the paper would benefit from presenting that transparently rather than letting readers infer it themselves.

>> **Reply:** We have added explicit reporting of the incremental gain from topological features in the Results section.

Comment 9: Page 18–19: The authors claim the model supports “early identification” of high-risk patients. I think the revised response adequately addresses this concern by introducing sequential prediction time points, but the manuscript should make this framing unmistakable. I recommend that the authors revise the wording wherever necessary so that “early” is understood as earlier dynamic risk updating during the early postoperative course, not prediction from baseline alone. This is now defensible, but it should be phrased with precision.

>> **Reply:** We have systematically reviewed all instances of "early identification" and revised to clarify the intended meaning.

Comment 10: Page 25–27: The authors discuss limitations and ethics, including bias, transparency, and integration into clinical workflows. I appreciate these additions. I still think the manuscript should state more explicitly that, despite encouraging subgroup

consistency, the sample size is too limited for definitive fairness conclusions. The current wording risks sounding slightly more reassuring than the subgroup counts justify, especially in smaller categories such as cystic fibrosis or single-lung transplant recipients. A more cautious sentence here would improve the paper.

>> **Reply:** We have revised the discussion to include a more cautious interpretation of subgroup findings, particularly for smaller subgroups.

Comment 11: Page 26: The authors claim "The integration of persistent homology with machine learning has recently shown promise in diverse fields including ECG analysis, paleontological image classification, geoscience porosity mapping, small peptides signalling in plants, and renewable energy monitoring." I appreciate that the authors did respond to the request for broader applicability. However, this part of the revision still feels underdeveloped relative to the importance of the point. The paragraph currently reads as a brief list rather than a genuine synthesis of how and why the methodological ideas transfer across domains. I encourage the authors to expand this into a more cohesive paragraph that explicitly links the shared methodological challenges: high-dimensional structured data, limited sample sizes, nonlinear manifold structure, and the need for interpretable prediction in high-stakes settings.

>> **Reply:** This is a constructive points. We have substantially expanded the broader applicability paragraph to provide a genuine synthesis of the shared methodological challenges and how topological feature engineering addresses them across domains.

Comment 12: Page 26: The authors added only a small subset of the recommended cross-domain references. Since this broader-applicability discussion was one of the key conceptual requests in the prior review round, I encourage the authors to strengthen it further with a better-balanced and more representative citation set. In particular, the revised paper would benefit from citing a wider group of recent cross-domain studies that make the transferability argument concrete rather than symbolic. I do not think this needs to become an extensive literature review, but it should be more than a short list of three or four examples.

>> **Reply:** We have expanded the reference set to include a broader range of recent cross-domain studies that demonstrate the transferability of topological and related methodological approaches.

Comment 13: Page 26–27: I do not think the current revision fully addresses the broader-positioning point, and I encourage the authors to strengthen this part more deliberately. At present, the added discussion still reads as a brief acknowledgment that similar methods exist elsewhere, rather than a persuasive explanation of why the methodological logic of this paper matters beyond lung transplantation. This is important because one of the manuscript's real strengths is not only its specific clinical application, but its potential relevance to a wider class of problems involving high-dimensional, structured, time-evolving data, limited sample sizes, nonlinear latent organization, and the need for interpretable prediction in high-stakes settings. In that sense, the paper would be much better positioned if the authors explicitly framed their

contribution not simply as a transplant-risk model, but as an example of how topological feature engineering can enrich predictive learning when conventional representations may miss geometry, trajectory structure, or clinically meaningful shape information. That broader framing would give the work stronger interdisciplinary value and make the novelty feel more durable.

I therefore strongly encourage the authors to expand this paragraph into a more cohesive discussion supported by a richer and better-balanced citation set. In particular, the current revision would benefit from citing a wider range of recent studies showing that similar representation-learning challenges arise across physiological signal analysis, scientific imaging, geoscience, porous-media modeling, wireless engineering, and other compact scientific-ML domains. Examples that would fit naturally here include the ECG-focused survey by Ansari et al. in Artificial Intelligence Review (2025), which would help connect the manuscript's signal-structure perspective to physiological diagnostics; the paleontology survey by Yaqoob et al. in Artificial Intelligence Review (2025), which shows how sparse and highly structured scientific patterns can be learned meaningfully outside medicine; "MicroCrystalNet" (IEEE Access, 2025), which is relevant as a lightweight and explainable architecture for microstructural scientific data; "PoreViT: Automated pore typing in carbonate rocks using vision transformers and neighborhood features" (Computers & Geosciences, 2025), which similarly addresses structured spatial representations in scientific classification; "FluidNet-Lite: Lightweight convolutional neural network for pore-scale modeling of multiphase flow in heterogeneous porous media" (Advances in Water Resources, 2025), which is a strong example of compact surrogate modeling in complex scientific systems; and "Prediction of Indoor Wireless Coverage from 3D Floor Plans Using Deep Convolutional Neural Networks" (Proc. IEEE LCN, 2021), which demonstrates how learned representations can replace or complement computationally expensive modeling in engineering prediction tasks. I would also encourage the authors to add further external references beyond this immediate citation circle, for example work on physics-informed machine learning for building-performance simulation and work on mobile-network coverage prediction using multimodal deep learning and semantic segmentation, because these would help make the broader applicability argument look balanced rather than self-contained.

My point here is not merely that the manuscript should cite more papers. Rather, I think the authors should use these references to argue more convincingly that the paper's core methodological value lies in showing how topology-aware representations can support prediction in data-limited, high-stakes domains where ordinary tabular summaries may fail to capture latent structure. That is a much stronger and more compelling position than presenting the work as a narrowly specialized transplant application. If the authors develop this argument properly, the paper will not only read as more mature and better situated in the literature, but will also have clearer appeal to a broader scientific and engineering audience.

>> **Reply:** We have substantially revised the broader positioning to frame our work as an exemplar of a generalizable methodological approach. The expanded paragraph (shown in response to Comment 11) now explicitly articulates this framing and is supported by

a richer set of cross-domain examples.

Comment 14: Page 27: The authors claim “This study introduces a topological attention-based predictive model for post-transplantation mortality.” I find “topological attention” still slightly imprecise and potentially confusing, because the method does not appear to implement an attention mechanism in the neural-network sense either. I encourage the authors to consider whether “topological feature engineering” or “topological feature-based prediction model” would be the cleanest and most transparent terminology, especially in the conclusion where readers look for a final statement of what was actually built.

>> **Reply:** We thank the reviewer for catching this remaining terminology issue. We have corrected the conclusion to use accurate terminology.

Comment 15: Page 27 and Data Availability: I appreciate that the authors acted quickly and responsibly in removing the original repository and replacing it with synthetic data and documentation after the privacy concern was raised. This was the correct response. For completeness, I encourage the authors to ensure that the final manuscript’s Data Availability Statement fully matches the revised privacy-preserving access model and does not contain any residual wording suggesting unrestricted public access to real patient data.

>> **Reply:** We have reviewed the Data Availability Statement and confirmed that it accurately reflects the current privacy-preserving access model.

Comment 16: Throughout the manuscript: The writing is improved, but I still encourage one last careful editorial pass for concision and terminology consistency. Most of the major clarity issues have been fixed, yet a few sections still read more like responses to reviewers than polished final prose. The manuscript is now much closer to publishable form, and a final tightening of phrasing, especially around novelty, timing, and broader applicability, would help it read as a cohesive final article rather than a revised draft.

>> **Reply:** We have conducted a thorough editorial review of the entire manuscript, focusing on:

- Eliminating language that reads as responses to reviewers
- Ensuring consistent terminology throughout
- Improving concision and flow
- Verifying that all figure references are correct
- Checking that all supplemental materials are properly cited

Specific revisions include:

- Streamlined methods section descriptions
- Removed redundant phrases
- Unified terminology ("topological feature engineering" throughout)
- Improved transitions between sections
- Verified all cross-references to figures, tables, and supplements

---

7. PLOS authors have the option to publish the peer review history of their article (what does this mean?). If published, this will include your full peer review and any attached files.

Do you want your identity to be public for this peer review? If you choose “no”, your identity will remain anonymous but your review may still be made public.

For information about this choice, including consent withdrawal, please see our Privacy Policy.

Reviewer #1: None
Reviewer #3: No
